# Supplementary material for: Strand‐specific, high‐resolution mapping of modified RNA polymerase II
Source: Mol Syst Biol. 2016 Jun 10;12(6):874. doi: 10.15252/msb.20166869 (PMC4915518; doi:10.15252/msb.20166869)
Supplement: Supplementary file 1 — Expanded View Figures PDF [file MSB-12-874-s001.pdf]

Expanded View Figures

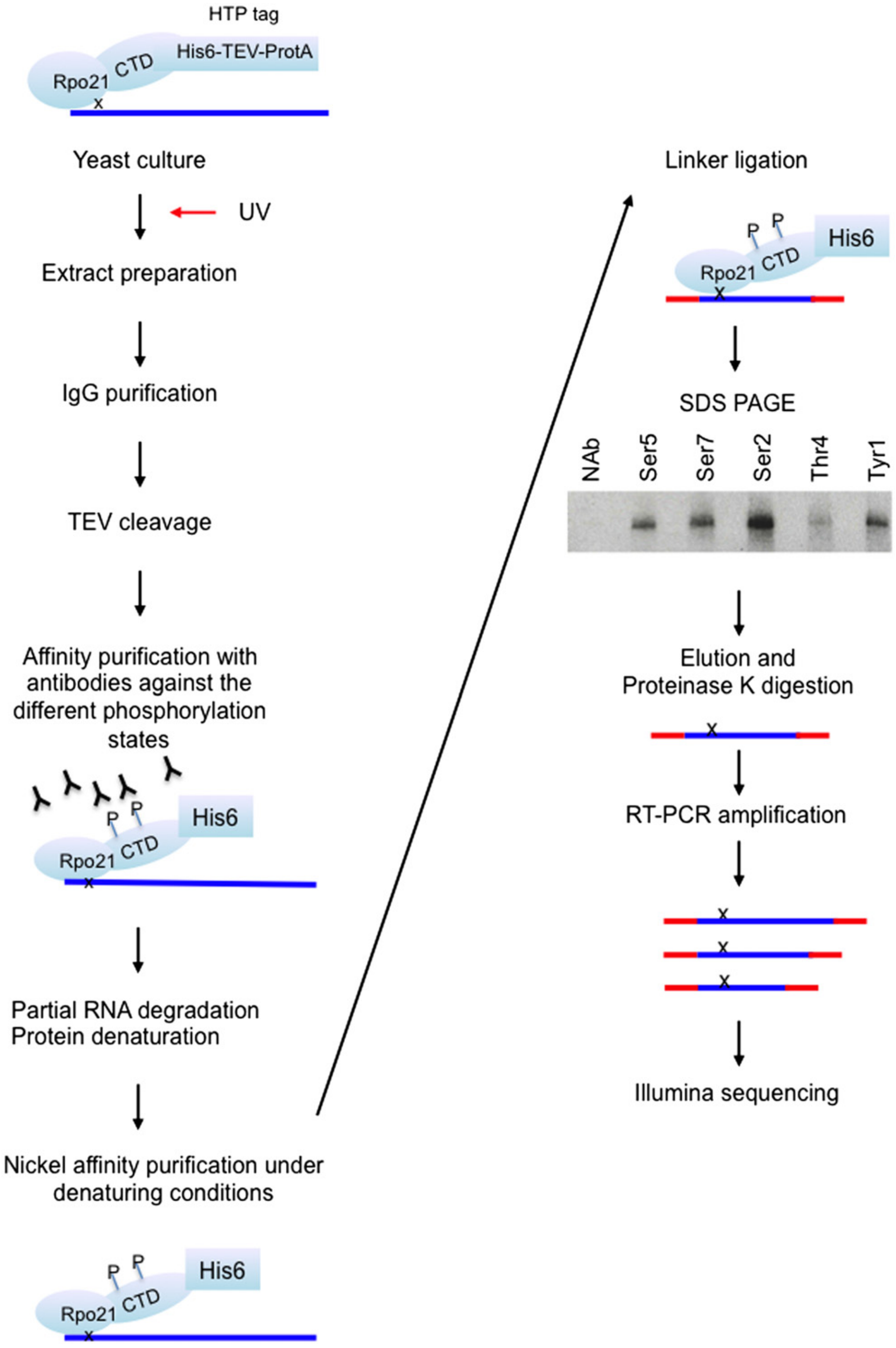

Figure EV1. Rpo21 crosslinking and purification during mCRAC.
The schematic indicates the major steps in the mCRAC protocol. The inset gel shows two replicates of the gel purification of Rpo21, with antibodies against the modifications indicated or a no antibody control (NAb), visualized by autoradiography of the 5′ [^{32}P] labeled, crosslinked RNA.

© 2016 The Authors

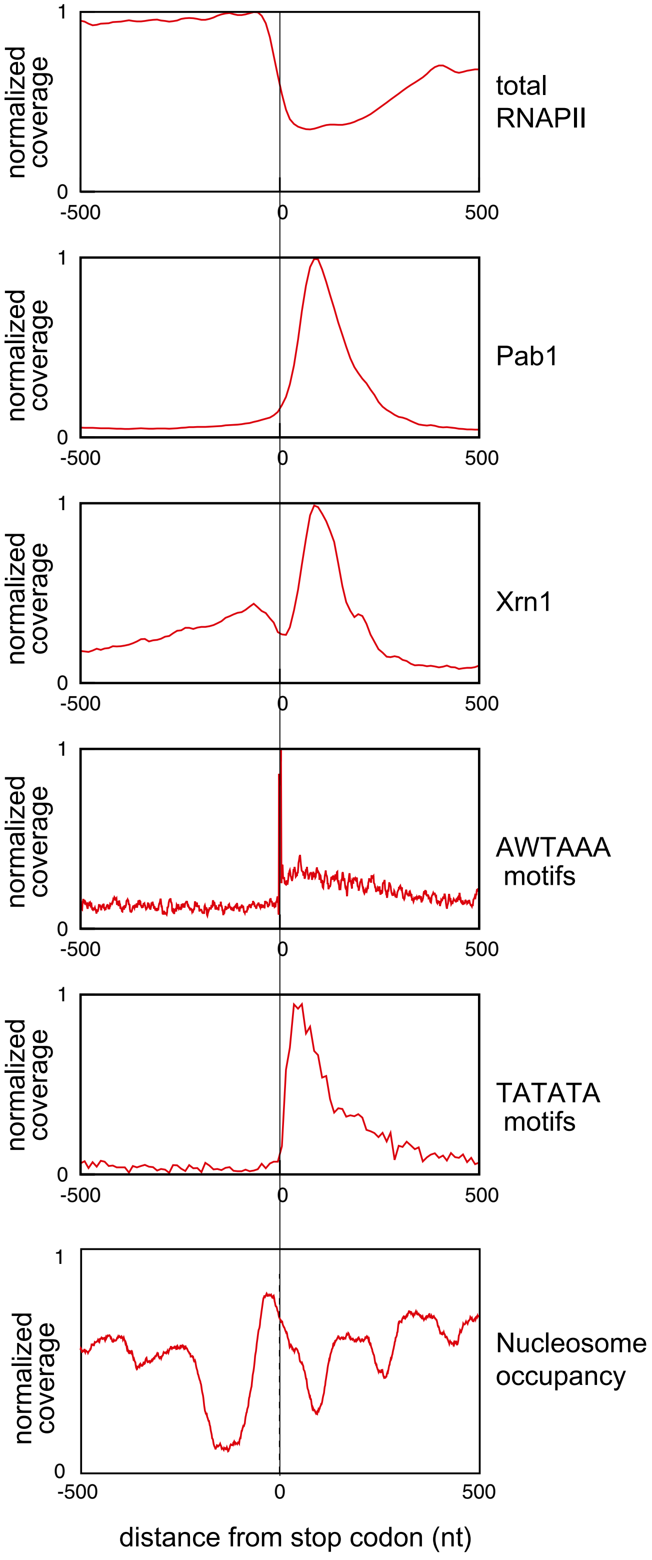

Figure EV2. Distribution of RNAPII with respect to gene features.
RNAPII signals drop around the position of the translation termination codon. The graphs show the site of the termination codon as 0 plus 500 nt flanking regions. The density of crosslinking over all protein-coding genes is shown for Rpo21 (total RNAPII), as well as the poly(A)-binding protein Pab1 and the cytoplasmic degradation factor Xrn1 (data from Tuck & Tollervey, 2013). The frequency of polyadenylation signals, AWTAAA and TATATA, are also indicated. Nucleosome distribution: The curve shows the fraction of transcripts where a nucleosome matches the corresponding position.

© 2016 The Authors

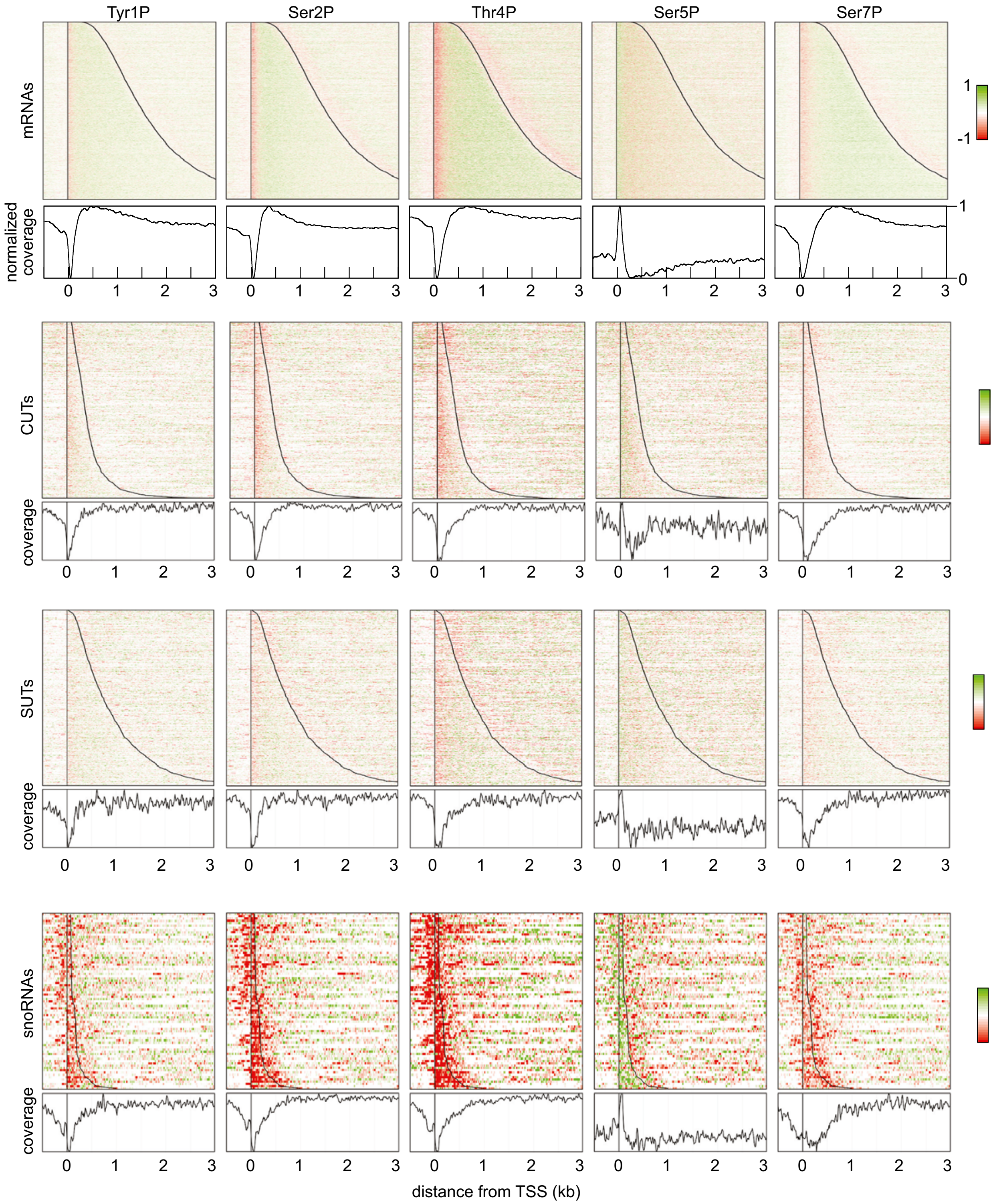

Figure EV3. Distribution of RNAPII phosphorylation across mRNAs and non-coding RNA genes.
Distribution of RNAPII phosphorylation as in Fig 3A, across all protein-coding genes, compared to the SUT, CUT, and snoRNA classes of ncRNA. Red color indicates depletion, and green color indicates enrichment of phosphorylation relative to total RNAPII. The graph below each panel shows a metagene analysis of RNAPII phosphorylation enrichment for all genes in each class.

 © 2016 The Authors

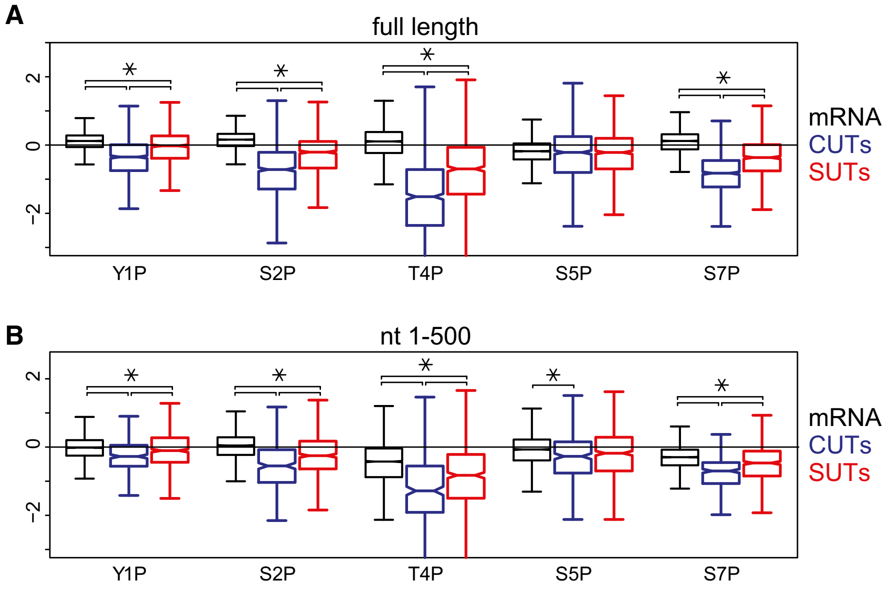

Figure EV4. Comparison of distributions of phosphorylation enrichment between different transcript classes.

A Distributions of phosphorylation enrichment on individual mRNAs, CUTs, and SUTs. The boxes show the median and interquartile range of phosphorylation enrichment scores for mRNAs (*N* = 5,171), CUTs (*N* = 925), and SUTs (*N* = 847).

B Distributions of phosphorylation enrichment in the first 500 nt of mRNAs, CUTs, and SUTs. Only genes with length greater than 500 nt were analyzed. The boxes show the median and interquartile range of phosphorylation enrichment scores for mRNAs (*N* = 5,040), CUTs (*N* = 294), and SUTs (*N* = 563).

Data information: The whiskers indicate 1.5 times the interquartile range, and the asterisks indicate statistical significance ($P < 0.01$, Wilcoxon test with Bonferroni correction, $N = 30$).

© 2016 The Authors

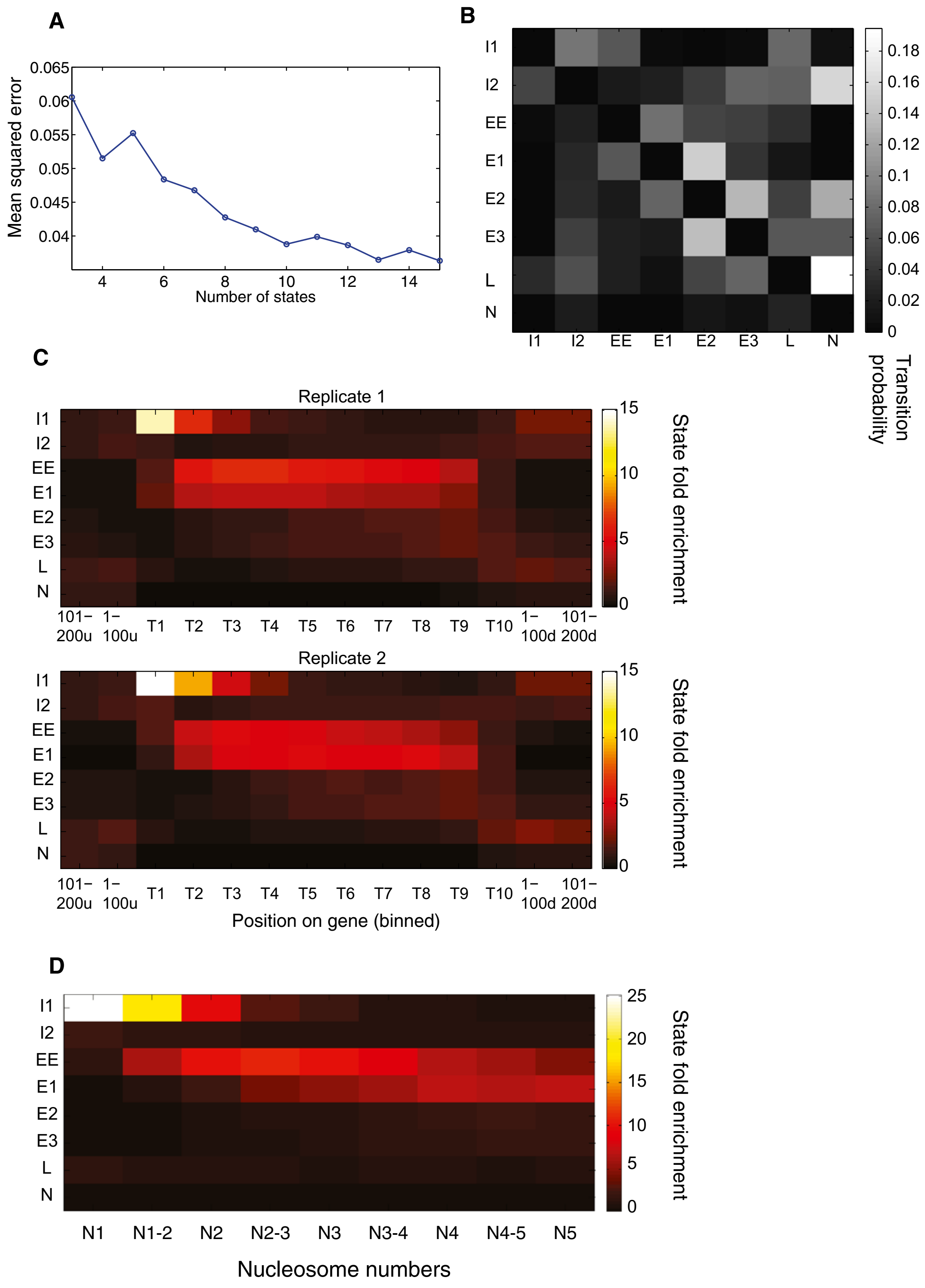

Figure EV5. HMM transition matrix, reproducibility of results, and state enrichment analysis.

A Plot showing the mean squared error with respect to the number of states in the HMM.

B Learned state transition matrix for 8-state HMM. Each entry (i,j) of the matrix shows the probability of transitioning from hidden state i to j at any position along the genome.

C State distributions across first quartile of protein-coding genes from two independent mCRAC analyses.

D State distributions across first quartile of protein coding relative to the positions of nucleosomes 1–5 (N1–N5) on these genes.

© 2016 The Authors

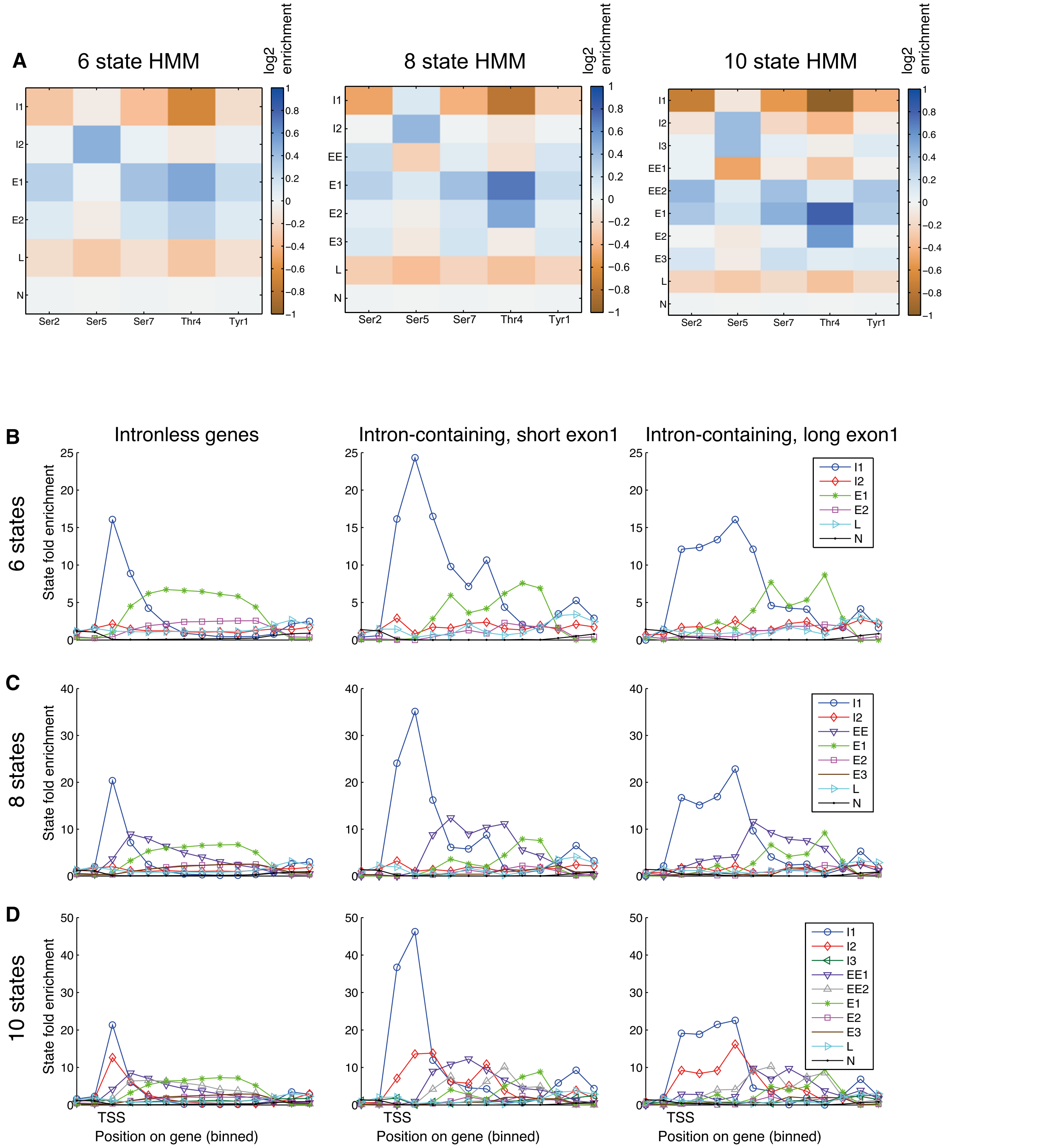

Figure EV6. Comparison of the effects of different state numbers in the HMM.

A Emission matrices for 6-, 8-, and 10-state HMM models.

B–D (B) 6-state HMM model; (C) 8-state HMM model; (D) 10-state HMM model. In each panel, the left graph (I) shows the top quartile of protein-coding genes, the middle graph (II) shows spliced genes with short exon 1 regions (< 100 nt) and the right graph (III) shows spliced genes with long exon 1 regions (> 100 nt). In each model, preferential initiation and elongation states are clearly resolved. In all models, the initiation state is clearly extended further 3′ on intron-containing genes.

© 2016 The Authors

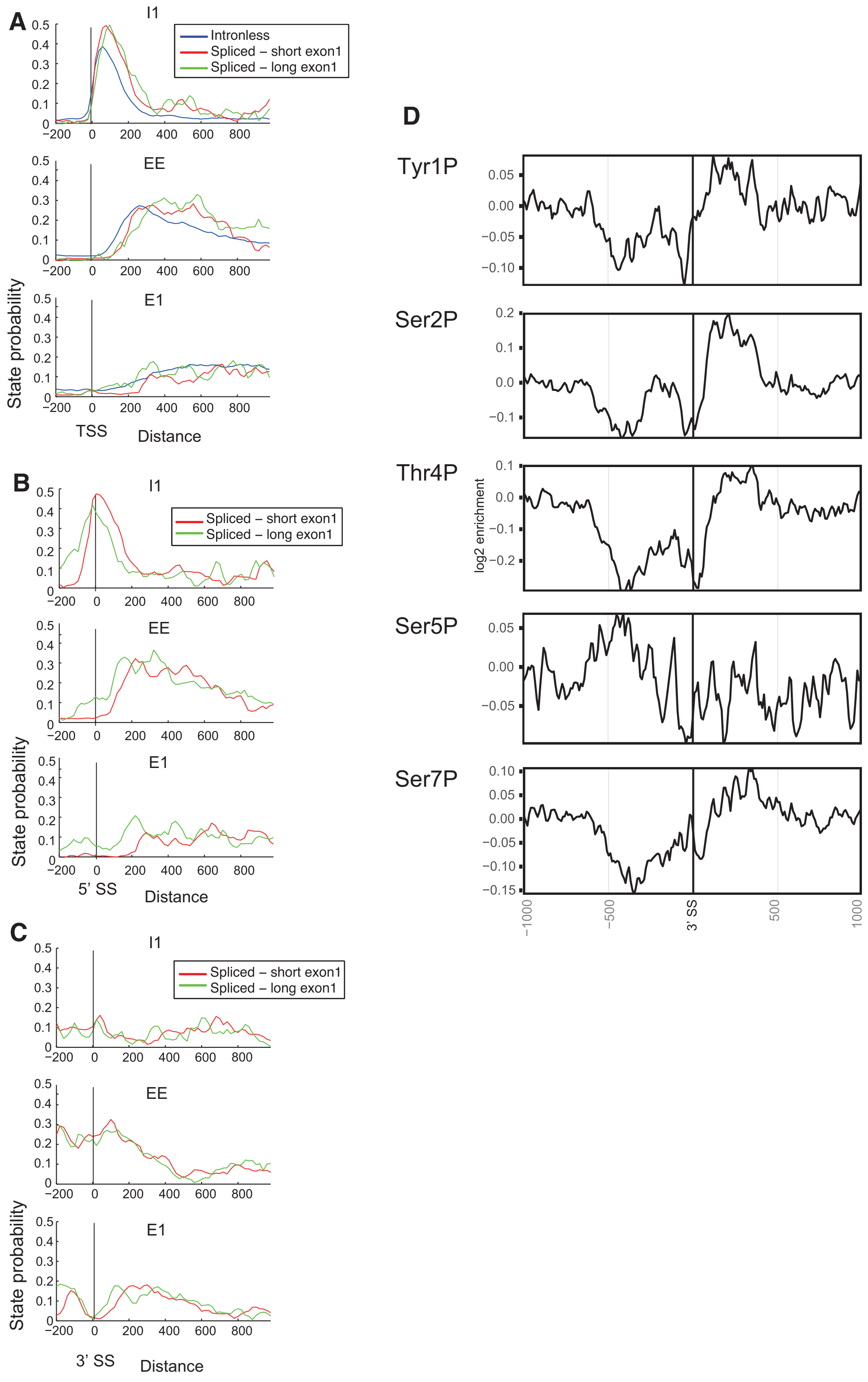

Figure EV7. The presence of an intron is associated with displacement of phosphorylation state boundaries.

- A Average state probabilities shown individually for each state over protein-coding genes lacking an intron (intronless) or with short (< 100 nt) or long (> 100 nt) exon 1 regions, aligned by the transcription start site (TSS).
- B, C Average state probabilities shown individually for each state over protein-coding genes with short (< 100 nt) or long (> 100 nt) exon 1 regions, aligned by 5′ splice site (5′SS; panel B) or 3′ splice site (3′SS; panel C).
- D Distribution of phosphorylated RNAPII relative to the 3′ splice site (3′SS) in intron-containing genes.

 © 2016 The Authors
