# Supplementary material for: Strand‐specific, high‐resolution mapping of modified RNA polymerase II
Source: Mol Syst Biol. 2016 Jun 10;12(6):874. doi: 10.15252/msb.20166869 (PMC4915518; doi:10.15252/msb.20166869)
Supplement: Supplementary file 2 — Table EV1 [file MSB-12-874-s002.docx]

**Table EV1. Enrichment of phosphorylation on different transcript classes.**

Statistical analysis of distribution of phosphorylated forms of RNAPII on mRNAs, CUTs and SUTs. Data taken from graphs shown in Fig. 3DF, and analyzed in Fig. EV2B and C.

| RNA class^a^ | log2 enrichment  mean (std deviation)^b^ | RNA class | log2 enrichment  mean (std deviation) | p-value^c^ | p-value corrected^d^ |
| --- | --- | --- | --- | --- | --- |
| Entire mRNA, CUT and SUT transcripts | | | | | |
| Y1P_mRNA | 0.108330 (0.293421) | **Y1P_CUTs** | -0.369198 (0.636960) | 5.84E-151 | 1.75E-149 |
| Y1P_mRNA | 0.108330 (0.293421) | **Y1P_SUTs** | -0.073583 (0.584660) | 2.59E-24 | 7.78E-23 |
| Y1P_CUTs | -0.369198 (0.636960) | **Y1P_SUTs** | -0.073583 (0.584660) | 7.64E-28 | 2.29E-26 |
| S2P_mRNA | 0.131748 (0.337171) | **S2P_CUTs** | -0.755633 (0.803765) | 1.81E-264 | 5.44E-263 |
| S2P_mRNA | 0.131748 (0.337171) | **S2P_SUTs** | -0.300945 (0.694874) | 8.49E-102 | 2.55E-100 |
| S2P_CUTs | -0.755633 (0.803765) | **S2P_SUTs** | -0.300945 (0.694874) | 1.97E-38 | 5.92E-37 |
| T4P_mRNA | 0.025236 (0.560475) | **T4P_CUTs** | -1.531818 (1.240829) | 1.99E-303 | 5.97E-302 |
| T4P_mRNA | 0.025236 (0.560475) | **T4P_SUTs** | -0.830410 (1.090475) | 2.88E-139 | 8.64E-138 |
| T4P_CUTs | -1.531818 (1.240829) | **T4P_SUTs** | -0.830410 (1.090475) | 1.67E-38 | 5.01E-37 |
| S5P_mRNA | -0.198000 (0.422760) | **S5P_CUTs** | -0.348851 (0.973127) | 1.14E-02 | 3.41E-01 |
| S5P_mRNA | -0.198000 (0.422760) | **S5P_SUTs** | -0.264181 (0.867845) | 9.55E-02 | 2.87E+00 |
| S5P_CUTs | -0.348851 (0.973127) | **S5P_SUTs** | -0.264181 (0.867845) | 3.46E-01 | 1.04E+01 |
| S7P_mRNA | 0.077685 (0.352310) | **S7P_CUTs** | -0.814733 (0.649958) | 0.00E+00 | 0.00E+00 |
| S7P_mRNA | 0.077685 (0.352310) | **S7P_SUTs** | -0.363227 (0.591702) | 2.68E-120 | 8.04E-119 |
| S7P_CUTs | -0.814733 (0.649958) | **S7P_SUTs** | -0.363227 (0.591702) | 2.86E-51 | 8.58E-50 |
| Nucleotides 1-500 of mRNA, CUT and SUT transcripts longer than 500 nt | | | | | |
| Y1P_mRNA | -0.024790 (0.376846) | **Y1P_CUTs** | -0.259795 (0.537912) | 5.70E-18 | 1.71E-16 |
| Y1P_mRNA | -0.024790 (0.376846) | **Y1P_SUTs** | -0.090422 (0.578811) | 2.81E-04 | 8.44E-03 |
| Y1P_CUTs | -0.259795 (0.537912) | **Y1P_SUTs** | -0.090422 (0.578811) | 3.72E-05 | 1.11E-03 |
| S2P_mRNA | 0.010112 (0.442270) | **S2P_CUTs** | -0.560890 (0.735815) | 1.96E-50 | 5.88E-49 |
| S2P_mRNA | 0.010112 (0.442270) | **S2P_SUTs** | -0.265355 (0.718827) | 2.68E-26 | 8.03E-25 |
| S2P_CUTs | -0.560890 (0.735815) | **S2P_SUTs** | -0.265355 (0.718827) | 1.05E-09 | 3.15E-08 |
| T4P_mRNA | -0.508654 (0.732844) | **T4P_CUTs** | -1.329001 (1.088610) | 2.22E-46 | 6.65E-45 |
| T4P_mRNA | -0.508654 (0.732844) | **T4P_SUTs** | -0.919834 (1.096669) | 6.89E-23 | 2.07E-21 |
| T4P_CUTs | -1.329001 (1.088610) | **T4P_SUTs** | -0.919834 (1.096669) | 5.69E-08 | 1.71E-06 |
| S5P_mRNA | -0.118347 (0.551504) | **S5P_CUTs** | -0.348559 (0.808749) | 2.36E-08 | 7.08E-07 |
| S5P_mRNA | -0.118347 (0.551504) | **S5P_SUTs** | -0.274219 (0.944266) | 3.54E-04 | 1.06E-02 |
| S5P_CUTs | -0.348559 (0.808749) | **S5P_SUTs** | -0.274219 (0.944266) | 6.61E-02 | 1.98E+00 |
| S7P_mRNA | -0.305469 (0.381811) | **S7P_CUTs** | -0.731305 (0.542673) | 2.79E-51 | 8.38E-50 |
| S7P_mRNA | -0.305469 (0.381811) | **S7P_SUTs** | -0.452497 (0.629472) | 1.00E-15 | 3.01E-14 |
| S7P_CUTs | -0.731305 (0.542673) | **S7P_SUTs** | -0.452497 (0.629472) | 1.28E-10 | 3.83E-09 |

a, numbers of transcripts in each class: mRNA, N=5171; CUTs, N=925; SUTs, N=847; mRNA>500nt, N=5040; CUTs>500nt, N=294; SUTs>500nt, N=563;

b, the log2 enrichment for each transcript was calculated as log2((P+1)/(T+1)), where P is the number of reads per million (RPM) mapped to that transcript in the phosphorylated RNAPII mCRAC, and T is the RPM in the total RNAPII CRAC;

c, two-sided Wilcoxon test;

d, two-sided Wilcoxon test with Bonferroni correction (N=30).
