# Supplementary material for: Strand‐specific, high‐resolution mapping of modified RNA polymerase II
Source: Mol Syst Biol. 2016 Jun 10;12(6):874. doi: 10.15252/msb.20166869 (PMC4915518; doi:10.15252/msb.20166869)
Supplement: Supplementary file 3 — Table EV2 [file MSB-12-874-s003.docx]

**Table EV2. Jaccard indices comparing the HMM state sequences obtained from two independent mCRAC data sets (with positive and negative genomic strands presented separately).**

The Jaccard index for a state *i* is the ratio A/B, where A (resp. B) is the size of the intersection (resp. union) of the two sets of 20nt-windows that are predicted to be in state *i* in the two HMM sequences respectively

State | Strand + | Strand -

I1 | 0.3001 | 0.3012

I2 |    0.0659 | 0.0679

EE |    0.2585 | 0.2550

E1 |    0.1498 | 0.1447

E2 | 0.0864 | 0.0850

E3 | 0.1187 | 0.1217

L | 0.1050 | 0.1076

N | 0.7419 | 0.7429
