# Supplementary material for: Strand‐specific, high‐resolution mapping of modified RNA polymerase II
Source: Mol Syst Biol. 2016 Jun 10;12(6):874. doi: 10.15252/msb.20166869 (PMC4915518; doi:10.15252/msb.20166869)
Supplement: Supplementary file 4 — Code EV1 [file MSB-12-874-s004.zip › HMM_code/HMMall/KPMtools/asdemo.html]

asdemo


```
% ASORT
```

```
% a pedestrian NUMERICAL SORTER of ALPHANUMERIC data
```

```
 
```

```
% - create some data
```

```
          d = {
```

```
%         strings with one valid alphanumeric number
```

```
%         sorted numerically
```

```
                  '-inf'
```

```
                  'x-3.2e4y'
```

```
                  'f-1.4'
```

```
                  '-.1'
```

```
                  '+ .1d-2'
```

```
                  '.1'
```

```
                  'f.1'
```

```
                  'f -+1.4'
```

```
                  'f.2'
```

```
                  'f.3'
```

```
                  'f.10'
```

```
                  'f.11'
```

```
                  '+inf'
```

```
                  ' -nan'
```

```
                  '+ nan'
```

```
                  'nan'
```

```
%         strings with many numbers or invalid/ambiguous numbers
```

```
%         sorted in ascii dictionary order
```

```
                  ' nan nan'
```

```
                  '+ .1e-.2'
```

```
                  '-1 2'
```

```
                  'Z12e12ez'
```

```
                  'inf -inf'
```

```
                  's.3TT.4'
```

```
                  'z12e12ez'
```

```
%         strings without numbers
```

```
%         sorted in ascii dictionary order
```

```
                  ' . .. '
```

```
                  '.'
```

```
                  '...'
```

```
                  '.b a.'
```

```
                  'a string'
```

```
                  'a. .b'
```

```
          };
```

```
%   ... and scramble it...
```

```
          rand('seed',10);
```

```
          d=d(randperm(numel(d)));
```

```
 
```

```
% - run ASORT with
```

```
%   verbose output:              <-v>
```

```
%   keep additional results:     <-d>
```

```
          o=asort(d,'-v','-d');
```

```
% - or
```

```
%         p=asort(char(d),'-v','-d');
```

```
 
```

```
    'INPUT'       'ASCII SORT'    'NUM SORT'             'NUM READ'        
```

```
    '...'         ' -nan'         '--- NUMERICAL'        '--- NUMBERS'     
```

```
    '+ .1e-.2'    ' . .. '        '-inf'                 [             -Inf]
```

```
    '.1'          ' nan nan'      'x-3.2e4y'             [           -32000]
```

```
    '.b a.'       '+ .1d-2'       'f-1.4'                [             -1.4]
```

```
    '-inf'        '+ .1e-.2'      '-.1'                  [             -0.1]
```

```
    'f.1'         '+ nan'         '+ .1d-2'              [            0.001]
```

```
    ' -nan'       '+inf'          '.1'                   [              0.1]
```

```
    '-1 2'        '-.1'           'f.1'                  [                1]
```

```
    'nan'         '-1 2'          'f -+1.4'              [              1.4]
```

```
    'a string'    '-inf'          'f.2'                  [                2]
```

```
    'f.3'         '.'             'f.3'                  [                3]
```

```
    '+ .1d-2'     '...'           'f.10'                 [               10]
```

```
    'a. .b'       '.1'            'f.11'                 [               11]
```

```
    's.3TT.4'     '.b a.'         '+inf'                 [              Inf]
```

```
    '+inf'        'Z12e12ez'      ' -nan'                [              NaN]
```

```
    ' nan nan'    'a string'      '+ nan'                [              NaN]
```

```
    'f-1.4'       'a. .b'         'nan'                  [              NaN]
```

```
    'x-3.2e4y'    'f -+1.4'       '--- ASCII NUMBERS'    '--- ASCII NUMBERS'
```

```
    'inf -inf'    'f-1.4'         ' nan nan'             ' nan nan'        
```

```
    '+ nan'       'f.1'           '+ .1e-.2'             '+ .1e-.2'        
```

```
    'f.2'         'f.10'          '-1 2'                 '-1 2'            
```

```
    'f.11'        'f.11'          'Z12e12ez'             'Z12e12ez'        
```

```
    'Z12e12ez'    'f.2'           'inf -inf'             'inf -inf'        
```

```
    'z12e12ez'    'f.3'           's.3TT.4'              's.3TT.4'         
```

```
    'f -+1.4'     'inf -inf'      'z12e12ez'             'z12e12ez'        
```

```
    ' . .. '      'nan'           '--- ASCII STRINGS'    '--- ASCII STRINGS'
```

```
    'f.10'        's.3TT.4'       ' . .. '               ' . .. '          
```

```
    '.'           'x-3.2e4y'      '.'                    '.'               
```

```
    '-.1'         'z12e12ez'      '...'                  '...'             
```

```
    ' '           ' '             '.b a.'                '.b a.'           
```

```
    ' '           ' '             'a string'             'a string'        
```

```
    ' '           ' '             'a. .b'                'a. .b'           
```

```
 
```

```
% - show results
```

```
          o
```

```
o =
```

```
          magic: 'ASORT'
```

```
            ver: '30-Mar-2005 11:57:07'
```

```
           time: '30-Mar-2005 11:57:17'
```

```
        runtime: 0.047
```

```
    input_class: 'cell'
```

```
    input_msize: [29 1]
```

```
    input_bytes: 2038
```

```
    strng_class: 'char'
```

```
    strng_msize: [29 8]
```

```
    strng_bytes: 464
```

```
            anr: {16x1 cell}
```

```
            snr: {7x1 cell}
```

```
            str: {6x1 cell}
```

```
              c: [29x12 char]
```

```
              t: [29x12 logical]
```

```
              n: [16x12 char]
```

```
              d: [16x1 double]
```

```
 
```

```
          o.anr
```

```
ans =
```

```
    '-inf'
```

```
    'x-3.2e4y'
```

```
    'f-1.4'
```

```
    '-.1'
```

```
    '+ .1d-2'
```

```
    '.1'
```

```
    'f.1'
```

```
    'f -+1.4'
```

```
    'f.2'
```

```
    'f.3'
```

```
    'f.10'
```

```
    'f.11'
```

```
    '+inf'
```

```
    ' -nan'
```

```
    '+ nan'
```

```
    'nan'
```

```
 
```

```
% - run ASORT with no-space/template options
```

```
%   NOTE the impact of -w/-t order!
```

```
          s={'ff - 1','ff + 1','- 12'};
```

```
 
```

```
%   RAW
```

```
          o=asort(s,'-v');
```

```
 
```

```
    'INPUT'     'ASCII SORT'    'NUM SORT'             'NUM READ'        
```

```
    'ff - 1'    '- 12'          '--- NUMERICAL'        '--- NUMBERS'     
```

```
    'ff + 1'    'ff + 1'        'ff + 1'               [                1]
```

```
    '- 12'      'ff - 1'        'ff - 1'               [                1]
```

```
    ' '         ' '             '- 12'                 [               12]
```

```
    ' '         ' '             '--- ASCII NUMBERS'    '--- ASCII NUMBERS'
```

```
    ' '         ' '             '--- ASCII STRINGS'    '--- ASCII STRINGS'
```

```
 
```

```
%   remove SPACEs
```

```
          o=asort(s,'-v','-w');
```

```
 
```

```
    'INPUT'    'ASCII SORT'    'NUM SORT'             'NUM READ'        
```

```
    'ff-1'     '-12'           '--- NUMERICAL'        '--- NUMBERS'     
```

```
    'ff+1'     'ff+1'          '-12'                  [              -12]
```

```
    '-12'      'ff-1'          'ff-1'                 [               -1]
```

```
    ' '        ' '             'ff+1'                 [                1]
```

```
    ' '        ' '             '--- ASCII NUMBERS'    '--- ASCII NUMBERS'
```

```
    ' '        ' '             '--- ASCII STRINGS'    '--- ASCII STRINGS'
```

```
 
```

```
%   remove TEMPLATE(s)
```

```
          o=asort(s,'-v','-t',{'ff','1'});
```

```
 
```

```
    'INPUT'    'ASCII SORT'    'NUM SORT'             'NUM READ'        
```

```
    ' - '      ' + '           '--- NUMERICAL'        '--- NUMBERS'     
```

```
    ' + '      ' - '           '- 2'                  [                2]
```

```
    '- 2'      '- 2'           '--- ASCII NUMBERS'    '--- ASCII NUMBERS'
```

```
    ' '        ' '             '--- ASCII STRINGS'    '--- ASCII STRINGS'
```

```
    ' '        ' '             ' + '                  ' + '             
```

```
    ' '        ' '             ' - '                  ' - '             
```

```
 
```

```
%   remove TEMPLATE(s) than SPACEs
```

```
          o=asort(s,'-v','-t','1','-w');
```

```
 
```

```
    'INPUT'    'ASCII SORT'    'NUM SORT'             'NUM READ'        
```

```
    'ff-'      '-2'            '--- NUMERICAL'        '--- NUMBERS'     
```

```
    'ff+'      'ff+'           '-2'                   [               -2]
```

```
    '-2'       'ff-'           '--- ASCII NUMBERS'    '--- ASCII NUMBERS'
```

```
    ' '        ' '             '--- ASCII STRINGS'    '--- ASCII STRINGS'
```

```
    ' '        ' '             'ff+'                  'ff+'             
```

```
    ' '        ' '             'ff-'                  'ff-'             
```

```
 
```

```
%   remove SPACEs than TEMPLATE(s)
```

```
          o=asort(s,'-v','-w','-t','1');
```

```
 
```

```
    'INPUT'    'ASCII SORT'    'NUM SORT'             'NUM READ'        
```

```
    'ff- '     '- 2'           '--- NUMERICAL'        '--- NUMBERS'     
```

```
    'ff+ '     'ff+ '          '- 2'                  [                2]
```

```
    '- 2'      'ff- '          '--- ASCII NUMBERS'    '--- ASCII NUMBERS'
```

```
    ' '        ' '             '--- ASCII STRINGS'    '--- ASCII STRINGS'
```

```
    ' '        ' '             'ff+ '                 'ff+ '            
```

```
    ' '        ' '             'ff- '                 'ff- '            
```

```
 
```

Published with MATLAB® 7.0.4
