# Supplementary figures and images for: Strand‐specific, high‐resolution mapping of modified RNA polymerase II
Source: Mol Syst Biol. 2016 Jun 10;12(6):874. doi: 10.15252/msb.20166869 (PMC4915518; doi:10.15252/msb.20166869)

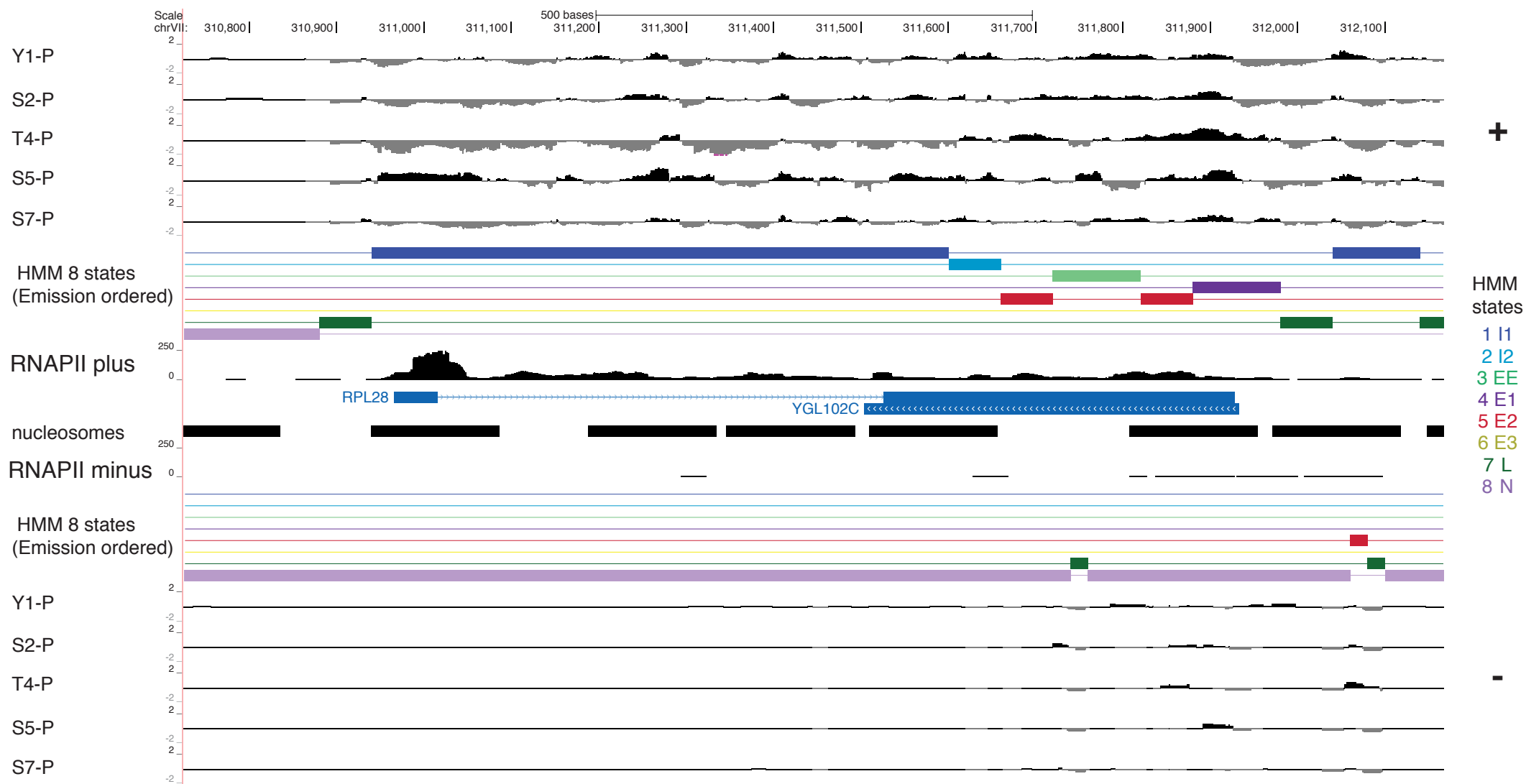

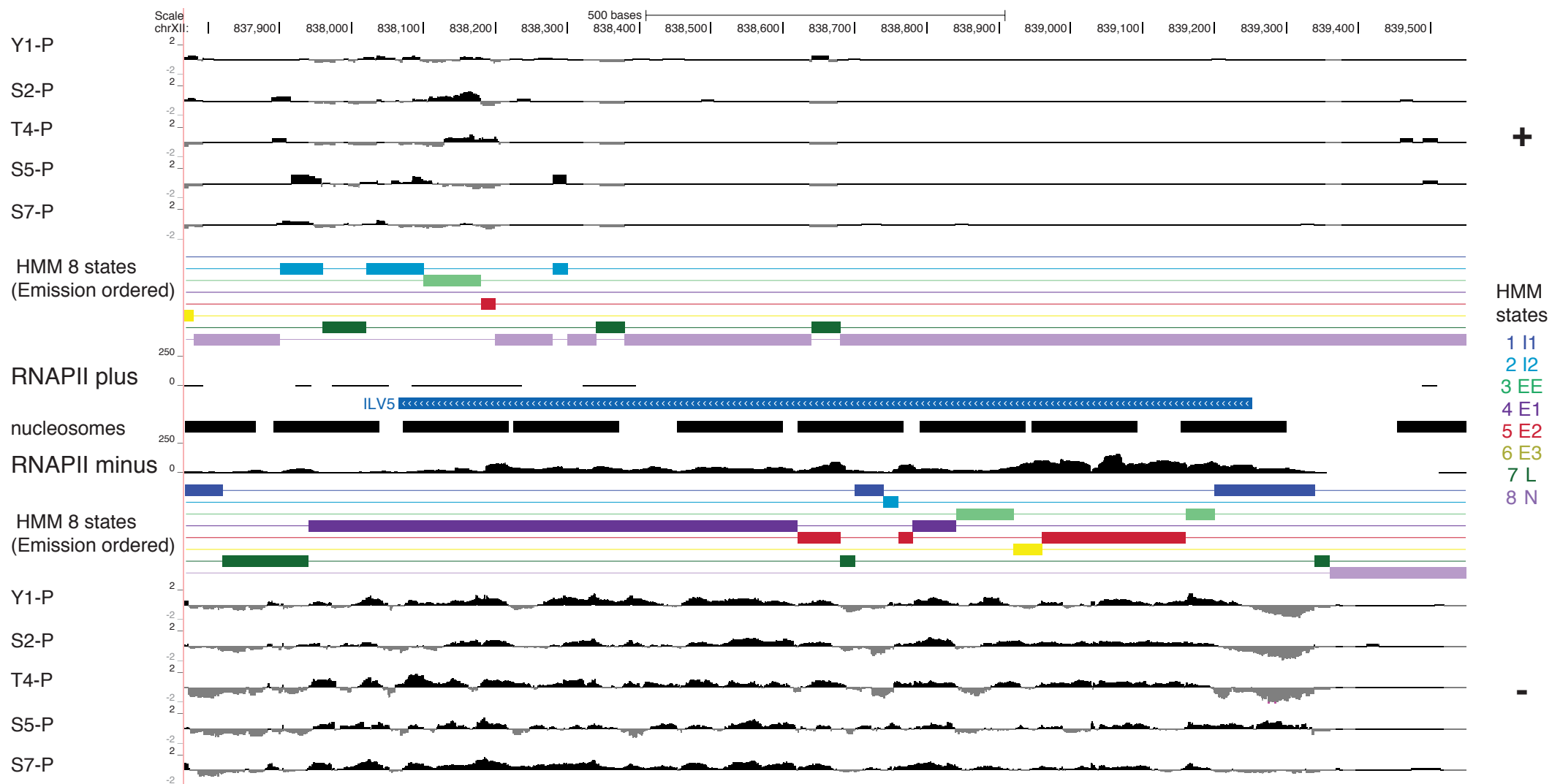

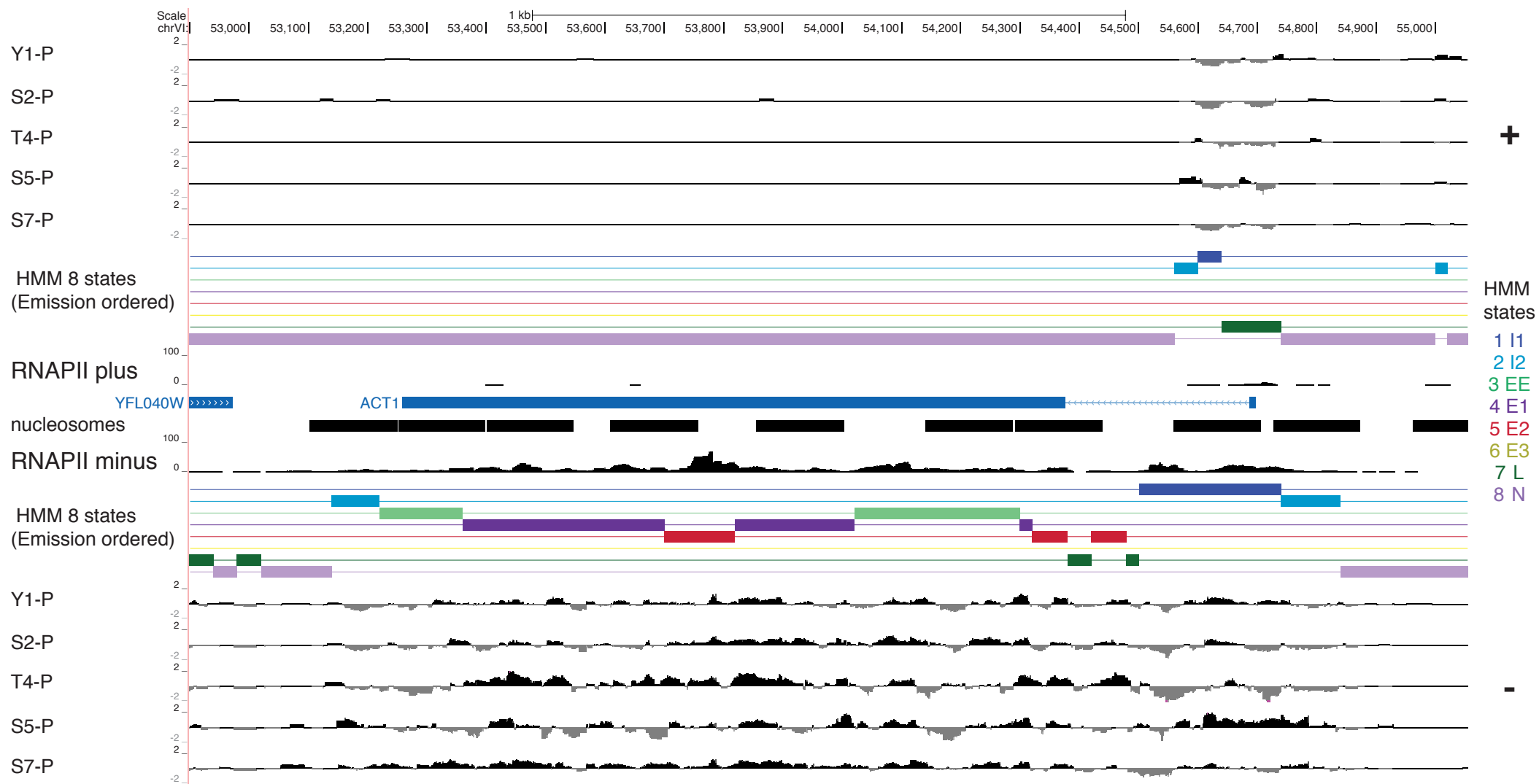

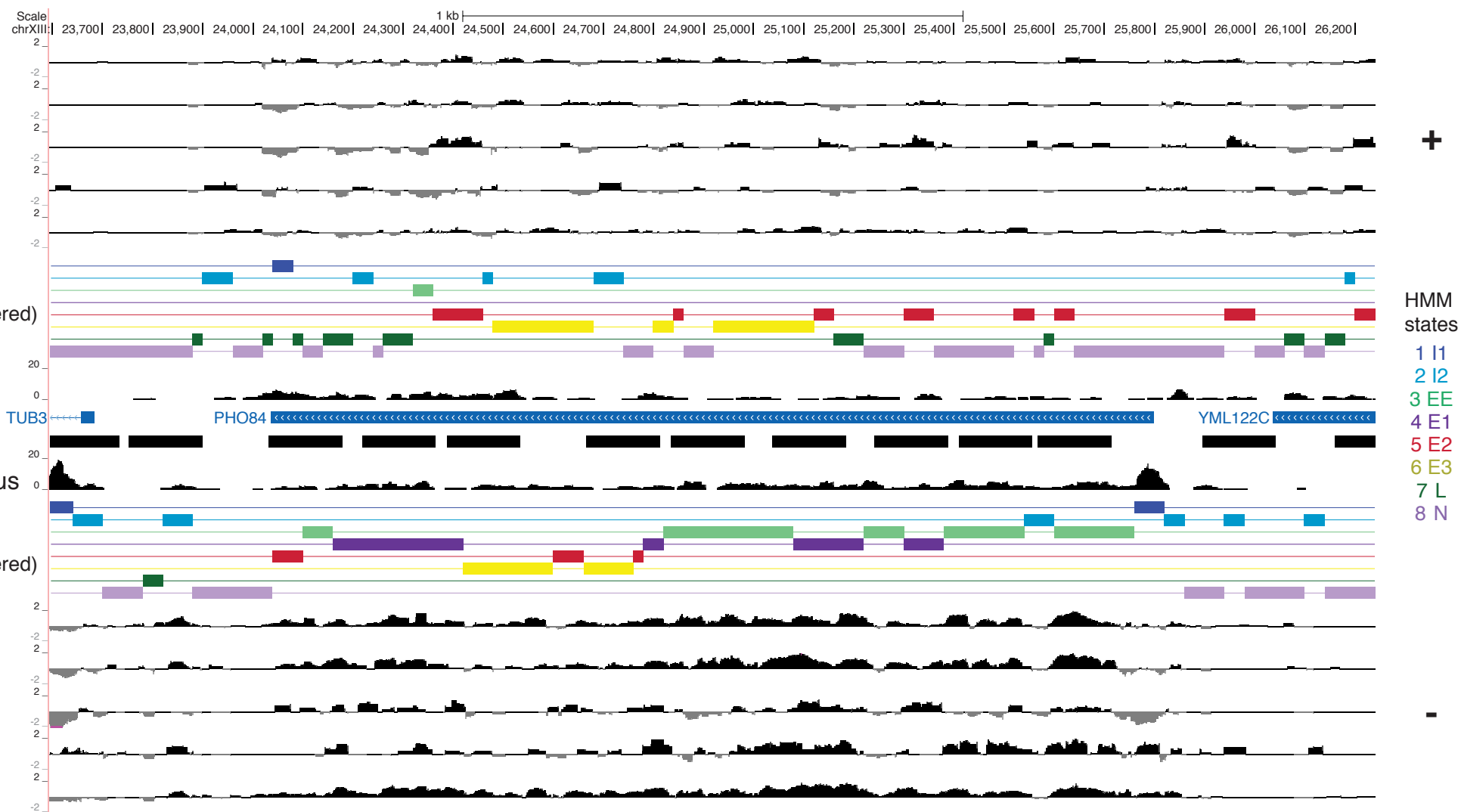

Supplement: Supplementary file 5 — Dataset EV1 [file MSB-12-874-s005.zip › Dataset_EV1/Dataset_EV1.pdf]
